# Surgical Experience and Complications in 50 Patients Treated with an Anular Closure Device Following Lumbar Discectomy

**DOI:** 10.1111/os.12495

**Published:** 2019-06-26

**Authors:** Ardeshir Ardeshiri, Larry E Miller, Michael Synowitz, Senol Jadik

**Affiliations:** ^1^ Department for Traumatology and Orthopaedics, Section for Spine Surgery Klinikum Itzehoe Itzehoe Germany; ^2^ Miller Scientific Consulting Asheville North Carolina USA; ^3^ Department for Neurosurgery University Hospital Kiel Kiel Germany

**Keywords:** Anular closure, Lumbar discectomy, Microdiscectomy, Reherniation

## Abstract

**Objective:**

To examine the results of an anular closure device for prevention of lumbar disc reherniation in daily routine practice.

**Methods:**

Fifty patients with large anular defects were treated with limited discectomy and a bone‐anchored anular closure device. The device physically occludes the defect in the anulus fibrosus and is intended for prevention of lumbar disc reherniation. Pain scores on a visual analogue scale, back function on the Oswestry Disability Index, and neurological status were noted. Symptomatic reherniation and reoperation rates were assessed at each follow‐up. Surgical findings and complications, device‐related and/or procedure‐related, were recorded. Follow‐up was 6, 12, 26, and 52 weeks.

**Results:**

Mean anular defect height/width was 4.6 mm/10.1 mm. The overall symptomatic reherniation and reoperation rate was 2%. During the 1‐year follow‐up period, mean back pain decreased from 43 to 8 (*P* < 0.001), leg pain decreased from 71 to 4 (*P* < 0.001), and the Oswestry Disability Index decreased from 46 to 5 (*P* < 0.001). Among 15 patients with preoperative neurological deficits, improvements in neurological function were noted in 14 (93%). There were no serious device‐related complications.

**Conclusions:**

The presented study shows promising early results in using the anular closure device. The procedure is safe with significantly fewer reherniations than for patients with large anular defects without anular closure. Further studies with longer follow‐up periods are warranted to prove these findings for long‐term outcomes.

## Introduction

Lumbar discectomy is one of the most frequently performed spine surgeries. Surgical treatment shows quicker improvement in pain compared to conservative treatment^1^, ^2^. The Spine Patient Outcomes Research Trial (SPORT) trial with 1244 cases, which is one of the biggest trials comparing conservative and surgical treatment of lumbar disc hernias, demonstrated that surgery is superior to conservative treatment after 3, 12, 24, and 48 months (SF‐36 Bodily Pain, Oswestry Disability Index)^3^.

Overall, discectomy results are good; however, 10%–30% of all surgically‐treated patients with lumbar disc herniations are unsatisfied^4^. One of the main reasons for dissatisfaction is recurrent disc hernias^5^, ^6^. Traditionally, an aggressive nucleus removal was performed during lumbar discectomy to reduce the risk of a recurrent herniation. However, aggressive nucleus removal has been reported to result in loss of disc height and resultant acceleration of degeneration, with the result of increasing back pain^7^, ^8^, ^9^. Therefore, a less aggressive technique with minimal nucleus removal, the so‐called limited discectomy, was established. This technique, where only the sequester is removed along with loose fragments near the defect, without performing a radical discectomy, was first described by Spengler^10^. In 2009, Watters^11^ compared 10 studies and pointed out that during follow‐up of at least 2 years, limited nucleus removal has a decreased risk of persistent back pain (7%–16%) compared to aggressive nucleus removal (19%–36%) but a higher risk of recurrent disc hernias (5%–18% *vs* 2%–10%). Similar findings were reported in a 2015 meta‐analysis by Ran *et al*.^9^ that included 12 studies. Ran *et al*. pointed out that limited discectomy is associated with less back pain than radical discectomy (weighted mean difference of 0.22 [95% CI 0.06–0.37, *P* < 0.05]) but higher reherniation rates (1.0%–21.2% *vs* 0%–10.5%).

In 2003, Carragee *et al*.^12^ published a landmark study with 187 patients demonstrating that anular defects wider than 6 mm had a reherniation rate of 27.3% compared to 1.1% in patients with very small or slit‐like anular defects. Kim *et al*.^13^ replicated these findings in 2015, pointing out that patients with lumbar disc hernias in L_5_/S_1_ with anular defects ≥6 mm had a reherniation rate of 18%. The study of McGirt *et al*.^14^ corroborates the correlation of large anular defects and higher reherniation rates.

Summing up the results of these studies, reherniation is a problem that is disproportionately focused on a smaller subset of patients with large anular defects. In addition, limited discectomy is a favorable technique to reduce the potential for subsequent back pain but bears a high risk of reherniation in this patient group^9^, ^11^. Surgical treatment of these patients creates a dilemma for the surgeon, who must decide intraoperatively to remove the nucleus radically or minimally.

**Figure 1:**
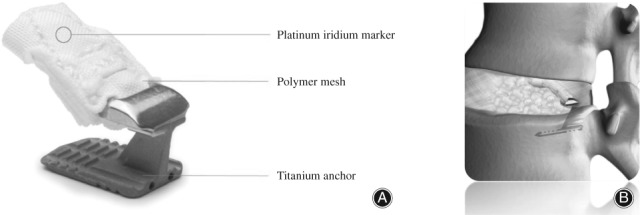
(A) Anular closure device (Barricaid) consisting of a titanium anchor and a polymer occlusion component with an iridium marker. (B) After implantation the mesh closes the anular defect and the implant is in a stable position by being anchored into the bone (figure is property of Intrinsic Therapeutics; publication permitted by Intrinsic Therapeutics).

To solve this dilemma, different techniques and implants have been investigated to close the anular defect after discectomy with the hope of reducing the reherniation rate without increasing the risk of more back pain resulting from aggressive nucleus removal. An anular closure device (ACD) was developed consisting of multiple layers of a polymer fabric and a titanium anchor (Barricaid, Intrinsic Therapeutics, Woburn, MA, USA). The dense fabric occludes the anular defect while the anchor, which is implanted into the vertebral body, ensures a stable position for the implant (Fig. 1). For the Barricaid device that is the subject of this study, little has been reported about the surgical results, including complications, under utilization in routine practice. Therefore, the present study was performed to evaluate the early safety results with a focus on intra‐operative and post‐operative complications of an ACD by a well‐controlled clinical trial.

## Materials and Methods

### *Inclusion and Exclusion Criteria*

Eligible patients were at least 18 years of age with a disc height ≥5 mm on preoperative MRI, and a large anular defect measuring ≥4 mm in height and ≥6 mm in width. Patients with significant osteoporosis, infections, tumors, or spondylolisthesis >1 were excluded.

### *Patient Population*

For this study, 50 consecutive patients with lumbar disc herniations treated with limited discectomy and anular closure were enrolled prospectively. All patients provided informed consent for treatment with the ACD. None of the included patients was excluded after surgery. Basic demographic data and the following data were recorded preoperatively: length of conservative care, prior lumbar surgery, neurological status, body mass index, back and leg pain scores (visual analogue scale, VAS), quality of life (Oswestry disability index, ODI) and disc height measured on MRI.

### *Surgery*

**Figure 2:**
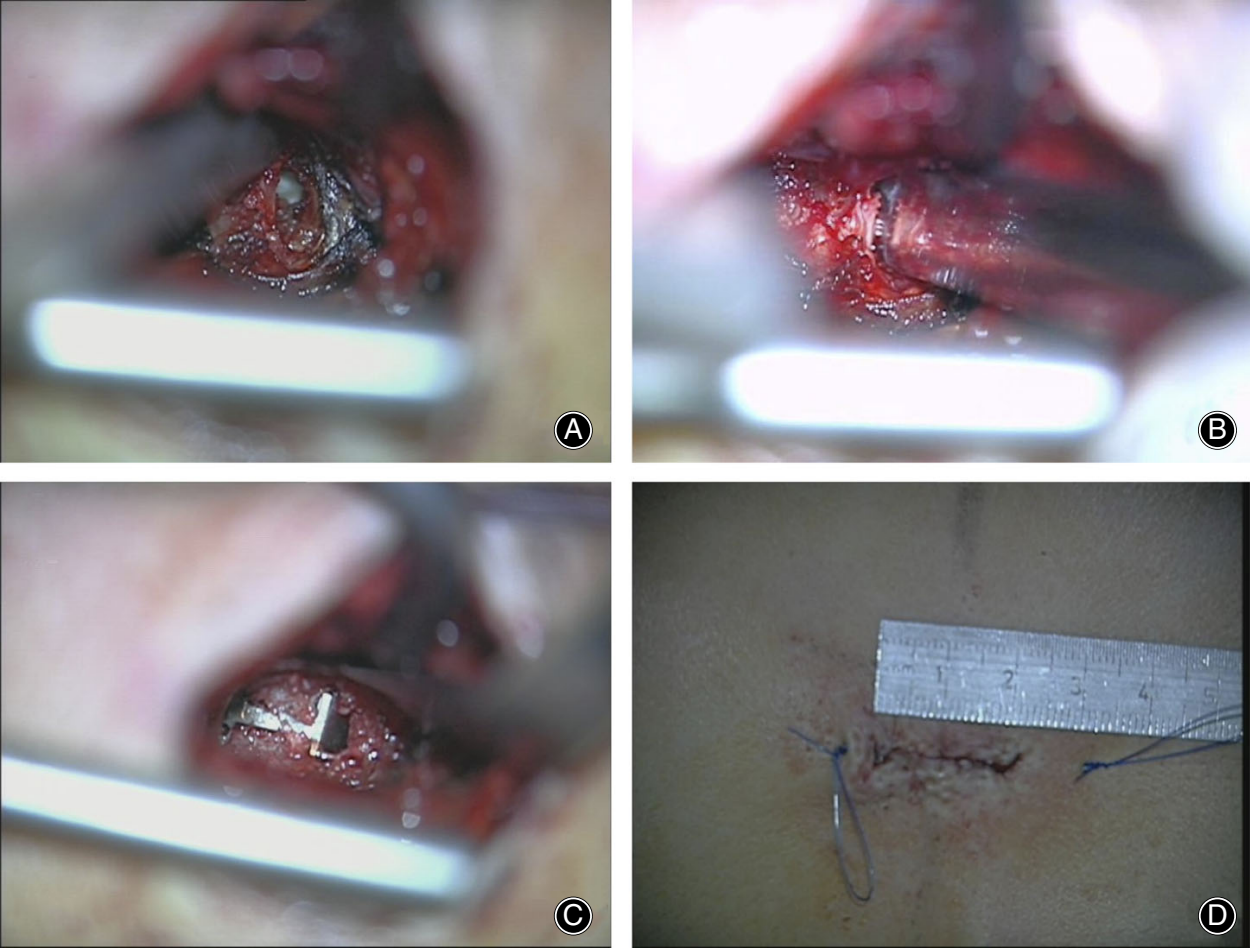
Intraoperative microscopic images. (A) Large anular defect is shown after limited discectomy. (B) The anular defect is measured by measuring tools. (C) Closure of the anular defect by the implant. (D) Small skin incisions are sufficient.

**Figure 3:**
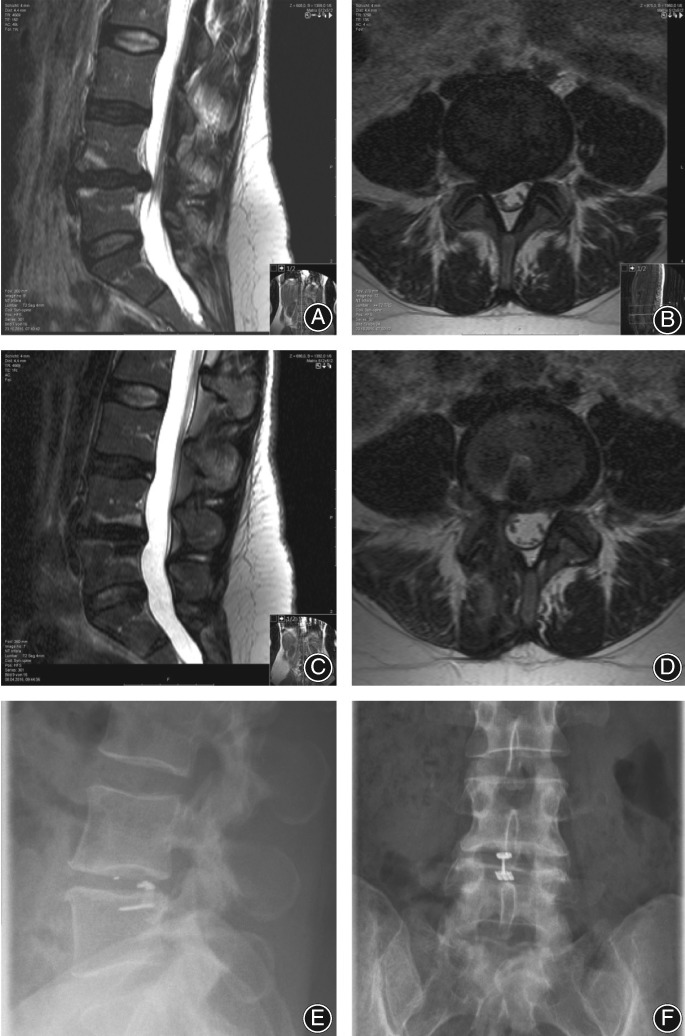
Lumbar disc hernia L_4/5_ right in a 40‐year old woman (A, B). The sequester (1.3 mL) was completely removed and the anular defect (4 mm height and 8 mm width) was closed by the anular closure device. (C, D) MRI 5 months postoperatively. Postoperative x‐rays show a correct position of the implant 2 mm ventral to the posterior wall of the vertebral body and anchor in L_5_ (E, F). The iridium marker of the occlusion component reveals a correct position of the device within the disc space (E).

All operations were performed under standard microsurgical conditions. The first step of the procedure involved limited discectomy according to Spengler^10^. The second step involved inspection of the anulus by selective incision of the posterior longitudinal ligament (PLL) to identify and visualize the anluar defect. For patients in which an anular defect was noted, the third step involved measurement of the defect height and width with special sizing instruments (Intrinsic Therapeutics, Woburn, MA, USA). If the defect height was 4–6 mm and width ≥6 mm, then the fourth step was to perform a sizing trial under fluoro control to assess whether the ACD could be implanted in the correct position and at the correct angle. Fifth, the ACD was implanted under fluoroscopic guidance by impacting the anchor into the vertebral body while the mesh was guided into the anular defect. Sixth, final X‐ray was taken to confirm accurate device placement. Finally, the surgical site was inspected and standard wound closure was performed (Figs 2 and 3).

Surgical data included defect height and width, amount of nucleus removal (measured in mL), surgical time, and surgical complications (associated and not associated with the implant).

### *Outcome Measures*

Patients were seen postoperatively at 6, 12, 26, and 52 weeks. Key outcome measures were back pain, leg pain, ODI, neurological status, and complications, including clinical signs of reherniation.

#### Functional Outcomes

Back pain and leg pain severity were each assessed on a 0–100 VAS, where higher scores represented more pain. ODI was measured on a 0–100 scale, where higher scores represented greater disability.

#### Neurological Status

Neurological status was assessed by physical examination and neurological deterioration was defined by a decrease in neurological status relative to the previous follow‐up visit.

#### Complications

All complications reported by a patient at each follow‐up visit, regardless of the relationship to the device or procedure, were recorded. The wound/scar was inspected at each visit to identify possible wound complications. Symptomatic reherniation was suspected if a patient presented with recurring radicular symptoms and a positive straight leg raise. In patients with suspected reherniation, X‐ray and MRI were performed to confirm or exclude the diagnosis. Patients with a worsening of VAS scores or ODI scores also underwent x‐ray and MRI to identify possible causes of clinical deterioration.

### *Statistical Analysis*

Baseline patient characteristics were reported as mean and standard deviation for continuous variables and count (percent) for categorical variables. Changes in back pain, leg pain, and ODI from baseline to 1 year were analyzed with a paired samples *t*‐test. Statistical significance was set at 0.05 and all tests were two‐sided. Data were analyzed using SPSS Statistics version 25 (IBM, Armonk, NY, USA).

## Results

### *General Results*

Twenty‐seven women and 23 men with an average age of 45.4 years were enrolled. All patients had significant conservative treatment duration with a mean symptom length of 8.3 months (range, 1–100 months). Mean body mass index of the 50 patients was 28.0 kg/m^2^. Three of the patients presented with a reherniation after primary surgery without an implant in another hospital and were treated with an ACD. One patient had a two‐level disc hernia and, therefore, overall, 51 segments were operated on in the present study. The most affected levels were L_4/5_ (26 cases) and L_5_/S_1_ (19 cases). Mean preoperative leg and back pain scores (VAS, 0–100) were 70.5 ± 33.2 and 43.4 ± 24.3, respectively. Mean ODI was 46.2/100 ± 16.3, and the disc height of the treated level was 6.3 mm (Table 1).

**Table 1:** Basic demographic data and intraoperative findings

Variables	Data
Male (*n*)	23
Female (*n*)	27
Age (years)	45.4 ± 14.0*
Body mass index (kg/m^2^)	28.0 ± 6.2*
Length of symptoms (months)	8.3 ± 15.7*
L_3_/_4_ (*n*)	5
L_4_/_5_ (*n*)	26
L_5_/S_1_ (L_5_/L_6_) (*n*)	20 (1/20 was L_5_/_6_)
Disc height (mm)	6.3 ± 1.4*
Removed nucleus (mL)	1.1 ± 0.7*
Defect height (mm)	4.6 ± 0.5*
Defect width (mm)	10.1 ± 1.6*
Surgical time (min)	52.2 ± 16.9*

* Values are expressed by mean ± standard deviation.

### *Intraoperative Findings*

Mean surgical time was 52.2 min. The removed nucleus amount measured 1.1 mL on average. The average anular defect height was 4.6 mm and width was 10.1 mm with a mean defect area of 46.5 mm^2^ (Table 1).The most used mesh size was 12 mm (38 levels in 37 patients). Nine patients were treated with a 10‐mm and four with an 8‐mm mesh.

In 2 patients we had small issues with the instruments as more force than normal was necessary to detach the applicator from the implant, leading to a small dural tear in 1 of these 2 cases. In 2 further patients the anchor could not be implanted into the attempted inferior vertebral body as the mesh missed the anular defect during implantation. In these cases, the ACD was inverted and could easily be implanted into the superior vertebral body. In another case, the ACD was placed an extra 2 mm dorsally. Nerve root injuries were considered as serious complications. Overall, there was no nerve root injury (either related or not related to the implant).

### *Functional Outcomes*

#### Visual Analogue Scale

Patients were seen at 6 (45 patients), 12 (47 patients), 26 (46 patients), and 52 weeks (45 patients) after surgery. Leg pain (VAS, 0–100) was 70.5 ± 24.5 preoperatively and decreased to 8.0 ± 14.8, 9.5 ± 21.1, 7.3 ± 16.0, and 4.4 ± 14.8 after 6, 12, 26, and 52 weeks, respectively. Back pain was 43.4 ± 33.5 preoperatively and decreased to 13.5 ± 19.0, 13.3 ± 22.1, 12.1 ± 20.8, and 8.0 ± 18.9 after 6, 12, 26, and 52 weeks, respectively.

#### Oswestry Disability Index

**Figure 4:**
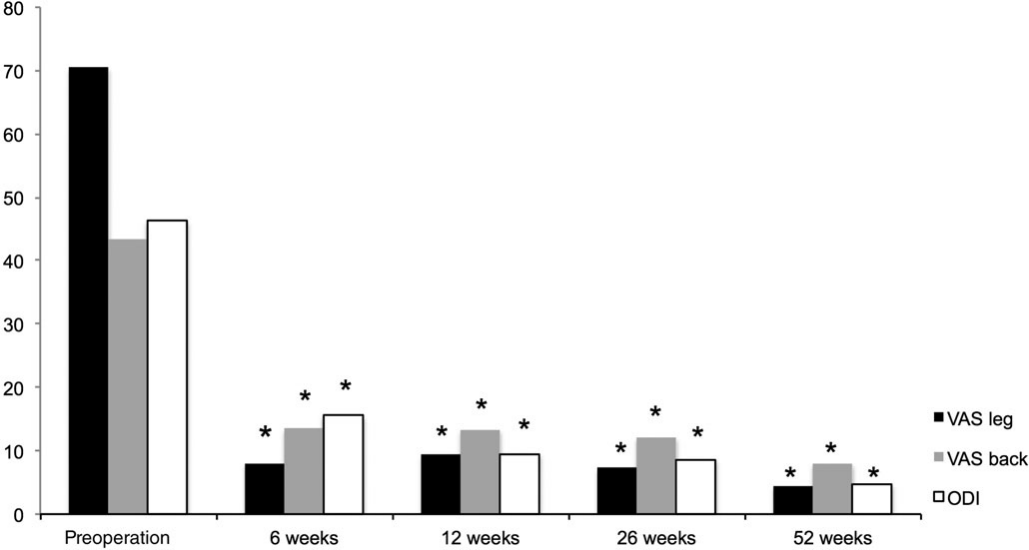
Preoperative and follow‐up data of leg/back pain (VAS) and Oswestry disability index (ODI) (*P < 0.0001 *vs* pre‐op).

Function and disability, measured by ODI, was 46.2 ± 16.5 before surgery and decreased to 4.7 ± 10.4 at 1‐year follow‐up (15.5 ± 16.7 after 6 weeks, 9.5 ± 16.1 after 3 months, and 8.6 ± 12.6 after 6 months). Pain scores and ODI reduced significantly (*P* < 0.0001) at all follow‐up time points (Fig. 4).

### *Neurological Status*

Neurologically, 15 of our 50 patients had a motor deficit due to the disc herniation before surgery, which improved in 14 (93.3%) cases (in 1 of these patients there was a slight worsening of the long toe extensor while the foot elevator improved postoperatively). In 1 patient there was no change in the preoperative motor deficit.

### *Complications*

There were no wound infections. We had 1 patient with reherniation (2.0%) 6 weeks after primary surgery who underwent reoperation. After reoperation the recovery course was uneventful. Another patient had a second surgery 8 months after the first surgery due to dislocation of the implant to dorsal. The reason was an improper first implantation (ACD was implanted at the posterior border of the vertebral body and not 2 mm ventral of it). During follow‐up none of the other patients had a reoperation.

## Discussion

The presented study consists of typical “real‐life” discectomy patients with a high rate of reherniation due to the large anular defect. The use of an anular closure device in these patients was safe and appears to substantially reduce the risk of recurrent herniation.

### *Pain Scores*

Six months after surgery VAS leg pain was reduced significantly, from 70.5 preoperatively to 4.4, and VAS back pain was reduced from 43.4 to 8.0. Lumbar discectomy studies showed consistent findings at 6 months: Peul^15^ had preoperative VAS pain scores of 67.2 (leg) and 33.8 (back) decreasing to 12.0 and 15.5, respectively. Similar findings were showed by Lequin^4^: VAS leg and back pain decreased 6 months postoperatively from values of approximately 80 and 60 to 20 and 10, respectively. Comparing the conventional discectomy patients’ results in the abovementioned studies with those for the technique of limited discectomy followed by anular closure, the presented study reveals that this technique results in the same positive effects on leg and back pain as conventional discectomy with the added benefit of mitigating risk of recurrent reherniation and repeat surgery.

### *Oswestry Disability Index (ODI) and Neurological Status*

The ODI is one of the instruments most frequently used to describe function and disability in patients requiring spine surgery. Before surgery, the presented patient population had a mean ODI of 46.2, which translates to significant disability in this study cohort, reducing to 4.7 at 12 months. In comparable studies, patients had similar results. Lequin^4^ reported values of approximately 60 falling to 12 after surgery. Carragee's^5^ patients recorded ODI scores of 52.7 preoperatively, decreasing to 17.4 at 12 months after surgery. The presented study with the use of an anular closure device shows no safety concerns related to disability and function compared to conventional microdiscectomy without anular closure.

Neurologically, 15 of our 50 patients had a motor deficit due to the disc herniation before surgery; 14 patients (93.3%) improved postoperatively (1 of them had a slight worsening of the long toe extensor while the functional important foot elevator improved) and no change was seen in 1 patient. In Yorimitsu's^16^ study 76.2% had a motor deficit preoperatively, while only 14.3% had a motor deficit at last follow‐up (mean 14.3 years). In a review from 2015^17^ concerning complications after lumbar disc surgery (depending on the surgical technique), neurological worsening postoperatively was 1.3%, 3.0%, and 1.6% for the open microsurgical, endoscopic, and percutaneous techniques, respectively. Overall, thorough comparison with the literature, our study reveals that anular closure poses no safety risk of neurological worsening.

### *Complications*

The excellent meta‐analysis of Shriver^17^ reviewed complication rates of the different surgical techniques (open microsurgical, endoscopic, and percutaneous) and divided complications into nine different types (nerve root injury, neurological worsening, medical complications such as thrombosis, surgical error, dural tear, hematoma, wound complication, recurrent disc hernia, and reoperation). The overall complication rate was 12.5%, 13.3%, and 10.8% for the three different techniques. General medical complication rates were 2.6%, 2.6%, and 0.0%. Wound complications between the three groups amounted to 2.1%, 1.2%, and 0.5% and dural tears occurred at a rate of 3.9%, 4.5%, and 0.0%. In the presented study, only one (2%) small dural tear was related to the anular closure device during removal of the implant applicator. Another complication was a dislocation of the implant due to an improper first implantation. The ACD should be implanted 2 mm ventral of the posterior border of the vertebral body, but in this patient the ACD was implanted at the level of the posterior border (not deep enough), leading to a dorsal dislocation. In the first 12 months postoperatively, no wound problems were noted. Therefore, in comparison to complication rates reported in the published literature, anular closure with the ACD implant revealed no higher complication rates than conventional microdiscectomy.

### *Reherniations*

Disc reherniations are both a clinical and a socioeconomic problem^18^, ^19^. Rates of reherniation deviate in the literature from 3%–18% to 7%–26%^5^, with an overall average of 10%^5^, ^20^. A patient with a reherniation is associated with additional costs of $26.953^18^. Clinically, reherniations challenge spine surgeons due to altered anatomy and scar tissue. In most cases, an aggressive discectomy is performed with the risk of persistent postoperative back pain, leading to chronic pain and the possibility of fusion surgery.

Carragee *et al*.^12^ found out that large anular defects result in a higher risk of reherniation, compared to smaller anular fissures, up to 27.3% after 2 years. Further predictive factors for reherniation are smoking, diabetes, and disc protrusions^21^. In the presented study, all patients were high‐risk patients for reherniation as all had anular defects ≥6 mm^12^ Only one of them (2.0%) had a reherniation during the follow‐up at 12 months. Cheng^22^ investigated in his study with 207 patients who had a reoperation after lumbar disc surgery; 127 of the patients were reoperated on due to reherniations and 63 (49.6%) of them had reoperations at 6 months postoperatively. At longer timepoints, 9.4% of them had a second surgery due to reherniation between 6 and 12 months, 34.6% between 1 and 5 years, and 6.3% between 5 and 9 years. Therefore, it can be assumed that during the follow‐up of 12 months more than half of the reherniations were noted. The reherniation rate of 2% after 12 months in the presented study is quite low, suggesting that the use of an anular closure might minimize the risk of reherniation in a subset of patients that are considered at a disproportionately high risk of recurrent disc herniation. These observations are reported from a study that was conducted outside of a well controlled clinical trial in a setting of normal daily practice.

### *Study and Method Limitations*

The presented study reveals that the use of an ACD in patients with large anular defects appears to reduce the risk of a reherniation significantly. Nevertheless, the study has some limitations which should be taken into account. The follow‐up period of 12 months is not long and the sample size (50 patients) is small. Furthermore, there is no control group and no routine imaging follow‐up was performed. Therefore, no data can be given concerning cost effectiveness relative to discectomy alone or to the frequency of mesh or implant migration. Finally, because eligible patients had large anular defects following lumbar discectomy, no conclusions can be drawn from this study on the efficacy of anular closure in small anular defects.

### *Conclusions*

Following lumbar disc surgery, reherniation rates are high in patients with large anular defects. While an anular closure device has been studied in a randomized superiority study, little has been reported about this kind of implant utilized within routine daily practice. The presented study demonstrates that the use of an anular closure device in this setting is safe with reduction of reherniation without higher complication rates or identified safety concerns. Further studies will be meaningful to prove these findings with long‐term follow‐up.
